# Association between physical activity and change in renal function in patients after acute myocardial infarction

**DOI:** 10.1371/journal.pone.0212100

**Published:** 2019-02-19

**Authors:** Toshimi Sato, Masahiro Kohzuki, Masahiro Ono, Mitsuru Muto, Taku Osugi, Keiichi Kawamura, Wakako Naganuma, Masayuki Sato, Namiko Shishito

**Affiliations:** 1 Department of Internal Medicine and Rehabilitation Science, Tohoku University Graduate School of Medicine, Sendai, Japan; 2 Department of Rehabilitation, Southern Tohoku General Hospital, Koriyama, Japan; 3 Department of Cardiology, Southern Tohoku General Hospital, Koriyama, Japan; 4 Department of Cardiology, Ohara General Hospital, Fukushima, Japan; International University of Health and Welfare, School of Medicine, JAPAN

## Abstract

**Background:**

Combined renal dysfunction worsens the subsequent prognosis in patients after acute myocardial infarction (AMI). Therefore, establishing a therapeutic modality to maintain or improve renal function in AMI patients is necessary. This study aimed to elucidate the association between physical activity level and change in renal function in such patients.

**Design:**

Prospective and observational study.

**Methods:**

We enrolled 41 patients (35 men; average age, 67.5 ± 12.6 years) after AMI onset. Blood biochemistry, urinalysis, and physical function tests were conducted at discharge and 3 months after discharge. Renal function was evaluated based on cystatin C based-estimated glomerular filtration rate (eGFRcys). The number of steps was recorded for 3 months post-discharge. Generalized estimating equations (GEE) was used to test the association between physical activity level and within-patient changes in eGFRcys.

**Results:**

Patients were stratified into low (n = 21; number of steps, 2335 ± 1219 steps/day) and high groups (n = 20; number of steps, 7102 ± 2365 steps/day). eGFRcys significantly increased from baseline to after 3 months in the high group (76.5 ± 13.8 to 83.2 ± 16.0 mL/min/1.73 m^2^, q = 0.004), whereas no significant change was observed in the low group (65.1 ± 15.9 to 62.2 ± 20.2 mL/min/1.73 m^2^, q = 0.125). Result of GEE adjusted for potential confounding variables showed a significant positive association between physical activity level and within-patient changes in eGFRcys (p = 0.003). Changes in eGFRcys was -2.9 mL/min/1.73 m^2^ among low group versus +6.7 mL/min/1.73 m^2^ among high group.

**Conclusions:**

Physical activity level was positively associated with changes in renal function, demonstrating that high physical activity may suppress renal function decline in patients after AMI.

## Introduction

Ischemic cardiac events, such as acute myocardial infarction (AMI), cause a decline in renal function [1]. Furthermore, in patients after AMI, combined renal dysfunction increases subsequent total mortality and cardiovascular death [2]. The progression of chronic kidney disease (CKD) worsens the success rate of percutaneous coronary intervention (PCI) and prognosis [3,4]. Accordingly, establishing a therapeutic modality to maintain or improve renal function in patients after AMI is important. In recent years, renal function has been recognized as a new target for exercise-based cardiac rehabilitation [5], and we previously demonstrated the renal-protective effects of chronic exercise in an experimental animal model [6]. Additionally, cardiac rehabilitation focusing mainly on supervised exercise therapy for AMI patients [7], or a group of patients with heart disease including those with AMI [8,9], was reported to be associated with maintaining and improving renal function. These reports suggest the possibility of kidney protection through exercise in AMI patients. However, in Japan, the rate of cardiac rehabilitation implementation for outpatients after AMI is very low [10,11]. In many cases, patients select remote exercise management, such as maintaining physical activity level in daily life and walking based on education by medical staff, such as doctors or physiotherapists. Therefore, elucidating whether physical activity level in the daily life of AMI patients has an effect on the changes in renal function is necessary, but there are no previous reports. Furthermore, most previous studies that examined the effect of exercise on renal function in AMI patients estimated renal function with serum creatinine, which depends on skeletal muscle mass [12]. Physical activity, including exercise, can change serum creatinine levels via changes in skeletal muscle mass. Therefore, the use of cystatin C, which is independent of skeletal muscle mass, is recommended when examining the effects of physical activity on renal function [13]. Accordingly, the aim of this study was to elucidate the association between physical activity level and changes in renal function in patients after AMI using cystatin C.

## Materials and methods

### Study design and patients

**Fig 1:**
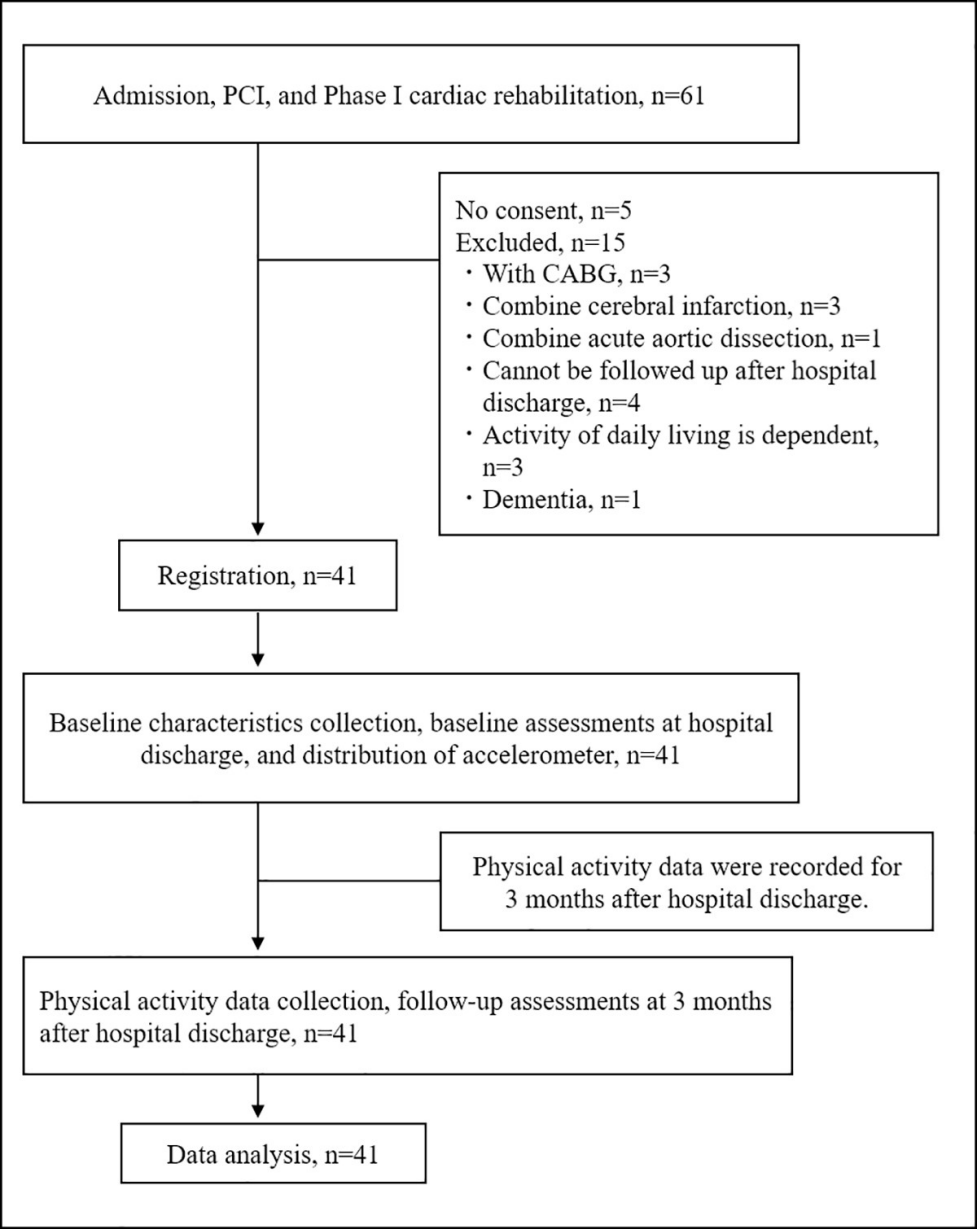
Study design. PCI, percutaneous coronary intervention; CABG, coronary artery bypass grafting.

This study was a prospective observational study, as shown in **Fig 1**. In this study, the follow-up period was decided to be 3 months with reference to a previous study [12] that examined the effect of exercise on renal function in AMI patients. Forty-one patients who were admitted to Southern Tohoku General Hospital from May 2017 to June 2018 due to AMI onset and who underwent PCI and cardiac rehabilitation during hospitalization were enrolled in the study. Exclusion criteria were as follows: refusal or inability to provide informed consent; follow-up not possible for 3 months after discharge; dependence on others for activities of daily living; receiving maintenance hemodialysis therapy; complicated by other acute diseases; underwent invasive treatments such as surgical operation during hospitalization or follow-up; and a diagnosis of dementia.

This study was conducted in accordance with the Helsinki Declaration. It was approved by the Ethics Committee of Tohoku University Graduate School of Medicine (Approval No. 2016‐1‐683) and the Ethics Committee of Southern Tohoku General Hospital (Approval No. D16‐14). All patients provided written informed consent after receiving an explanation about the purpose and protocol of the study.

### Baseline characteristics

Clinical information on age; sex; height; weight; body mass index; smoking history; employment status; comorbidities of CKD, diabetes mellitus, hypertension, and dyslipidemia; use of medications such as angiotensin-converting enzyme inhibitors, angiotensin receptor blockers, calcium-channel blockers, beta-blockers, and statins; Killip classification, peak value of serum creatine kinase; left ventricular ejection fraction on an echocardiogram; and Geriatric Depression Scale-15 score, in which a score of 5 or higher was defined as the presence of depressive symptoms [14], were collected from medical records or by interview at registration.

### Blood biochemistry and urinalysis

Blood biochemistry and urinalysis were performed at discharge and at 3 months after discharge. For blood biochemistry, B-type natriuretic peptide, hemoglobin, glucose, triglyceride, high-density lipoprotein (HDL) cholesterol, low-density lipoprotein (LDL) cholesterol, LDL/HDL ratio, cystatin C, creatinine, urea nitrogen, cystatin C-based estimated glomerular filtration rate (eGFRcys), and creatinine-based estimated glomerular filtration rate (eGFRcreat) were collected. For urinalysis, urine protein/creatinine ratio (UPCR) was collected. In this study, eGFRcys was used for the primary evaluation of renal function because serum creatinine levels, which depend on skeletal muscle mass [12,13], may be influenced by physical activity. GFR was estimated using the Japanese Society of Nephrology equation as follows:

eGFRcys (mL/min/1.73 m^2^) = 104 × Cystatin C^−1.019^ × 0.996^Age^ × 0.929 (if female)– 8 [15]

eGFRcreat (mL/min/1.73 m^2^) = 194 × Cre^−1.094^ × age^−0.287^ × 0.739 (if female) [16]

### Physical function tests and blood pressure measurement

Physical function tests, including the 6-min walk test (6MWT), handgrip strength test, and 30-second chair-stand test (CS30), were conducted at discharge and at 3 months after discharge. The 6MWT was conducted according to the American Thoracic Society recommendations [17]. The patients had to walk as far as possible. If they experienced shortness of breath and fatigue, they were allowed to reduce their walking speed and take a break as needed. After the break, they had to resume walking as soon as possible. Patients were informed of the elapsed time every minute by a physiotherapist. The test was conducted after a 10-min rest. Handgrip strength test was measured using a digital dynamometer (TKK 5101 Grip-D; Takei, Tokyo, Japan). This test was conducted according to the methods of a previous study [18]. It was performed with the right and left hands in turns. The highest strength values on the right and left sides were averaged and expressed as the absolute value (kg). The CS30 consists of standing up and sitting down from a chair as many times as possible within 30 s. A standard chair (with a seat height of 40 cm) without a backrest was used. The patients were initially seated on the chair with their back in an upright position. They were instructed to look straight forward and to rise after the “1, 2, 3, go” command at their own preferred speed with their arms folded across their chest [19]. Blood pressure measurements were conducted at discharge and 3 months after discharge. All tests and measurements were performed after a 10-min rest.

### Physical activity

To evaluate physical activity, we used an accelerometer with a tri-axial acceleration sensor (AM500N; Acos Co., Ltd, Nagano, Japan). The accelerometer was distributed to the patients at discharge. The patients were instructed to wear the accelerometer consistently for 3 months, except when bathing and sleeping. The patients were also instructed to record their daily number of steps in the recording notes for 3 months. The accelerometer can record the number of steps and duration of activity when the instrument detects acceleration greater than the preset threshold value of activity (metabolic equivalents [METs]). We set the value to 3.0 METs as the threshold of moderate intensity of physical activity [20]. Three months after discharge, the patients came to the hospital and physical activity data were transferred into a computer from the accelerometer via a reader (RC-S360/S; Acos Co., Ltd, Nagano, Japan). Subsequently, the average number of steps (steps/day) and duration of activity > 3.0 METs (min/day) for 7 consecutive days at 1 month after discharge and 3 months after discharge (total of 14 days) were calculated and recorded as the absolute values of both parameters. The 14 days were shown in the research manual and recording notes in advance, and patients were able to confirm the 14 days. When the patients performed activities that could not be measured by the accelerometer, such as cycling or swimming, or forgot to wear the accelerometer, these had to be indicated in the recording notes, and data on the relevant days had to be excluded from analysis. However, none of the patients fulfilled the exclusion provision during the 14 days. Then, we examined the maximum number of steps (steps/day), minimum number of steps (steps/day), and coefficient of variation of number of steps for each patient to confirm variations in physical activity in 14 days. We also examined the adherence rate of monitoring physical activity in 3 months and the main seasons of the monitoring from the recording notes.

### Statistical analysis

We calculated the median number of steps in all patients (4113 steps/day). Patients who walked >4113 steps/day and ≤4113 steps/day were classified into the high-activity group (high group) and low-activity groups (low group), respectively. Changes in eGFRcys (ΔeGFRcys) (mL/min/1.73 m^2^) were defined as the value obtained by subtracting the eGFRcys at discharge from the eGFRcys at 3 months after discharge and change in eGFRcreat (ΔeGFRcreat) was also calculated in the same manner [21,22]. Descriptive statistics were used to present baseline characteristics and several parameters related to physical activity in the high and low group. Clinical data including blood biochemistry, urinalysis, and physical function tests at baseline and both ΔeGFRcys and ΔeGFRcreat were compared between the two groups using an unpaired t-test or Wilcoxon signed-rank test. Clinical data at baseline and at 3 months after were compared within each group using a paired t-test or Wilcoxon signed-rank test.

As a main analysis, generalized estimating equations (GEE), which account for within-participant clustering, was used to test the longitudinal association between physical activity level and within-patient changes in eGFRcys adjusting for potential confounding variables. An unstructured working correlation structure was applied to adjust for within-patient correlation. As age [23,24], renal function [24], diabetes mellitus [25,26], hypertension [24,26], and dyslipidemia [26,27] have been reported as factors that can affect the progression of kidney disease, age, glucose, triglyceride, and systolic blood pressure at baseline were determined as covariates to assess the association. Interaction terms between physical activity level (high group, low group) and time (baseline, 3 months after) were included in a model to investigate the effects of physical activity level on change in eGFRcys during follow-up.

As a secondary analysis, to verify the difference between ΔeGFRcys and ΔeGFRcreat when verifying the continuous association between physical activity and change in renal function, we analyzed the correlations between the number of steps and ΔeGFRcys, or ΔeGFRcreat in all patients using Pearson’s correlation, and compared their correlations.

All analyses were performed using SPSS 21.0 for Windows (SPSS Inc. Chicago, IL). An alpha-level of 0.05 was considered for statistical significance. To reduce the risk of type I error in multiple comparisons in unpaired t-tests, paired t-tests, or Wilcoxon signed-rank tests on the clinical data at baseline and after 3 months in the low and high groups, we corrected the p-values to q-values for each of the tests, according to the false discovery rate (FDR) procedure [28]. The FDR threshold was set at q = 0.05.

## Results

### Physical activity

Forty-one patients were stratified as 21 patients in the low group (number of steps, 2335 ± 1219 steps/day; duration of activity >3.0 METs, 18.3 ± 11.0 min/day) and 20 patients in the high group (number of steps, 7102 ± 2365 steps/day; duration of activity >3.0 METs, 56.8 ± 24.1 min/day), as shown in **Table 1**. The adherence rate of monitoring physical activity in 3 months in the low group was 76.3% ± 31.7% and that in the high group was 91.3% ± 13.3%.

**Table 1:** Baseline characteristics and physical activity of low and high groups.

	All patients (n = 41)	Low group (n = 21)	High group (n = 20)
Age (years)	67.5 ± 12.6	72.0 ± 12.9	62.8 ± 10.8
Men	35 (85.4)	18 (85.7)	17 (85.0)
Height (cm)	163.4 ± 8.4	162.9 ± 8.0	164.0 ± 9.0
Weight (kg)	65.9 ± 12.5	64.4 ± 11.1	67.5 ± 13.9
BMI (kg/m^2^)	24.6 ± 3.7	24.2 ± 3.6	25.0 ± 3.8
Smoking			
Current	15 (36.6)	6 (28.6)	9 (45.0)
Former	15 (36.6)	8 (38.1)	7 (35.0)
Never	10 (24.4)	6 (28.6)	4 (20.0)
Employment	20 (48.0)	7 (33.3)	13 (65.0)
Comorbidities			
Chronic kidney disease	9 (22.0)	7 (33.3)	2 (10.0)
Diabetes mellitus	9 (22.0)	4 (19.0)	5 (25.0)
Hypertension	23 (56.1)	11 (52.4)	12 (60.0)
Dyslipidemia	23 (56.1)	7 (33.3)	16 (80.0)
Peak creatine kinase (IU/l)	2070 ± 1475	2056 ± 1422	2084 ± 1566
LVEF (%)	55.8 ± 12.8	50.4 ± 13.6	61.4 ± 9.3
Killip class			
Killip I	37 (90.2)	17 (81.0)	20 (100.0)
Killip II	2 (4.9)	2 (9.5)	0 (0.0)
Killip III	2 (4.9)	2 (9.5)	0 (0.0)
Killip IV	0 (0.0)	0 (0.0)	0 (0.0)
GDS-15 (points)	5 ± 3	5 ± 3	4 ± 3
GDS-15≥5 points	19 (46.3)	13 (61.9)	6 (30.0)
Medications			
ACEI/ARB	31 (75.6)	18 (85.7)	13 (65.0)
Calcium-channel blocker	4 (9.8)	3 (14.3)	1 (5.0)
Beta-blocker	25 (61.0)	14 (66.7)	11 (55.0)
Statin	34 (82.9)	16 (76.2)	18 (90.0)
Physical activity			
Average of number of steps (steps/day)	4660 ± 3036	2335 ± 1219	7102 ± 2365
Maximum number of steps (steps/day)	7828 ± 4880	4331 ± 2210	11499 ± 4159
Minimum number of steps (steps/day)	1984 ± 1610	953 ± 712	3066 ± 1586
Coefficient of variation of number of steps	0.4 ± 0.2	0.5 ± 0.3	0.3 ± 0.1
>3 METs (minutes/day)	37.9 ± 29.3	18.3 ± 11.0	56.8 ± 24.1
Adherence rate (%)	83.6 ± 25.4	76.3 ± 31.7	91.3 ± 13.3
Season of monitoring			
Spring (Mar, Apr, May)	8 (19.5)	3 (14.3)	5 (25.0)
Summer (Jun, Jul, Aug)	16 (39.0)	7 (33.3)	9 (45.0)
Fall (Sep, Oct, Nov)	11 (26.8)	6 (28.6)	5 (25.0)
Winter (Dec, Jan, Feb)	6 (14.6)	5 (23.8)	1 (5.0)

Data are presented as mean ± standard deviation or number (%). ACEI, angiotensin-converting enzyme inhibitor; Adherence rate, adherence rate of monitoring physical activity; ARB, angiotensin receptor blocker; BMI, body mass index; GDS-15, Geriatric Depression Scale-15; LVEF, left ventricular ejection fraction; >3 METs, the duration of activity >3 METs

### Baseline characteristics

**Table 1** shows the baseline characteristics of the low and high groups.

### Clinical data at baseline and after 3 months

**Table 2** shows the clinical data at baseline and after 3 months. At baseline, the high group had higher values of 6-min walk distance (6MWD), grip strength, and CS30, and lower value of BNP than the low group. In the high group, eGFRcys (76.5 ± 13.8 to 83.2 ± 16.0 mL/min/1.73 m^2^, q = 0.013), glucose, HDL cholesterol, 6MWD, systolic blood pressure, and diastolic blood pressure increased, and BNP, LDL cholesterol, LDL/HDL ratio, and cystatin C decreased from baseline to 3 months after. In contrast, in the low group, hemoglobin, glucose, triglyceride, HDL cholesterol, 6MWD, CS30, and systolic blood pressure increased, and BNP and LDL/HDL ratio decreased without significant changes in eGFRcys (65.1 ± 15.9 to 62.2 ± 20.2 mL/min/1.73 m^2^, q = 0.200). Based on the results of the comparison of changes in eGFR between the two groups, ΔeGFRcys in the high group was significantly higher than that in the low group (+6.7 ± 9.2 vs -2.9 ± 8.2, q = 0.009).

**Table 2:** Clinical data at baseline and after 3 months in the low and high groups.

	Low group (n = 21)			High group (n = 20)		
	Baseline	3 months after	q-value	Baseline	3 months after	q-value
**Blood laboratory data**						
eGFRcys (mL/min/1.73 m^2^)	65.1 ± 15.9	62.2 ± 20.2	0.200	76.5 ± 13.8	83.2 ± 16.0	0.013
ΔeGFRcys (mL/min/1.73 m2)	-	-2.9 ± 8.2	-	-	+6.7 ± 9.2 †	-
eGFRcreat (mL/min/1.73 m^2^)	61.1 ± 15.6	59.5 ± 17.6	0.397	69.7 ± 12.8	74.4 ± 13.9	0.068
ΔeGFRcreat (mL/min/1.73 m2)	-	-1.7 ± 7.9	-	-	+4.8 ± 10.0	-
BNP (pg/mL)	120.5 (68.8–268.2)	59.7 (42.1–109.2)	<0.001	45.2 (31.0–71.0) **	26.2 (14.6–36.0)	<0.001
Hemoglobin (g/dl)	13.1 ± 1.7	13.8 ± 1.8	0.027	14.3 ± 1.5	14.5 ± 1.2	0.525
Glucose (mg/dl)	104.2 ± 18.0	118.7 ± 28.9	0.041	107.0 ± 22.1	129.6 ± 40.1	0.018
Triglyceride (mg/dl)	107.1 ± 49.4	142.6 ± 70.1	0.048	133.7 ± 37.6	114.3 ± 40.4	0.1
HDL cholesterol (mg/dl)	40.7 ± 10.5	50.7 ± 7.9	0.002	40.4 ± 10.1	54.0 ± 13.2	<0.001
LDL cholesterol (mg/dl)	89.4 ± 19.6	84.1 ± 23.0	0.400	105.8 ± 31.6	85.3 ± 21.3	0.013
LDL/HDL ratio	2.3 ± 0.6	1.7 ± 0.4	0.001	2.7 ± 0.9	1.6 ± 0.5	<0.001
Cystatin C (mg/l)	1.10 ± 0.23	1.17 ± 0.29	0.060	0.97 ± 0.13	0.90 ± 0.14	0.012
Creatinine (mg/dl)	0.97 ± 0.20	1.00 ± 0.22	0.420	0.87 ± 0.16	0.83 ± 0.14	0.056
Blood urea nitrogen (mg/dl)	18.3 ± 5.7	17.5 ± 7.1	0.603	15.5 ± 3.2	15.7 ± 3.4	0.814
**Urine laboratory data**						
UPCR (g/gCr)	0.06 (0.05–0.13)	0.07 (0.05–0.10)	0.741	0.05 (0.04–0.07)	0.06 (0.04–0.10)	0.079
**Physical function**						
6-min walk distance (m)	329.5 ± 121.0	371.1 ± 129.6	0.016	473.1 ± 92.4 ***	521.6 ± 82.3	<0.001
Grip strength (kg)	26.5 ± 7.7	27.9 ± 8.4	0.250	33.1 ± 7.7 *	33.8 ± 7.9	0.274
CS30 (counts)	10 ± 3	13 ± 4	0.011	17 ± 5 ***	18 ± 4	0.250
**Resting blood pressure**						
Systolic blood pressure (mmHg)	118.2 ± 8.7	127.3 ± 13.4	0.013	114.7 ± 16.3	122.3 ± 18.0	0.034
Diastolic blood pressure (mmHg)	74.6 ± 7.6	78.5 ± 12.0	0.379	71.0 ± 9.1	77.6 ± 8.6	0.011

Data are presented as mean ± standard deviation, except for BNP and UPCR, which are expressed as medians (25th to 75th percentiles). All p-values were corrected to q-values using the false discovery rate procedure [28].

* q < 0.05, vs. low group at baseline

** q < 0.01, vs. low group at baseline

*** q < 0.001, vs. low group at baseline

† q < 0.01, vs. low group

BNP, B-type natriuretic peptide; Cr, creatinine; CS30, 30-second chair-stand test; eGFRcreat, creatinine-based estimated glomerular filtration rate; eGFRcys, cystatin C-based estimated glomerular filtration rate; UPCR, urine protein/creatinine ratio.

### Associations between physical activity and changes in eGFR

**Table 3** shows the results of the GEE analysis testing the longitudinal association between physical activity level and within-patient changes in eGFRcys. Both results of the crude model (p < 0.001) and adjusted model (p = 0.003) showed significant positive associations. Change in eGFRcys was -2.9 mL/min/1.73 m^2^ in the low group versus +6.7 mL/min/1.73 m^2^ in the high group. After adjustment for potential confounding variables, similar results were shown.

**Table 3:** Longitudinal associations between physical activity levels and within-patient changes in eGFRcys.

Physical activity level	Changes in eGFRcys (95% Confidence Interval)	
	Crude model	Adjusted model *
Low group (≤4113 steps/day)	-2.9 (-6.3 to 0.6)	-2.9 (-7.0 to 1.3)
High group (>4113 steps/day)	+6.7 (1.6 to 16.7)	+6.7 (1.4 to 12.0)
Wald Chi-square	19.2	13.9
p-value	< 0.001	0.003

*, Model adjusted for age, glucose, triglyceride, and systolic blood pressure at baseline. eGFRcys, cystatin C-based estimated glomerular filtration rate.

**Fig 2:**
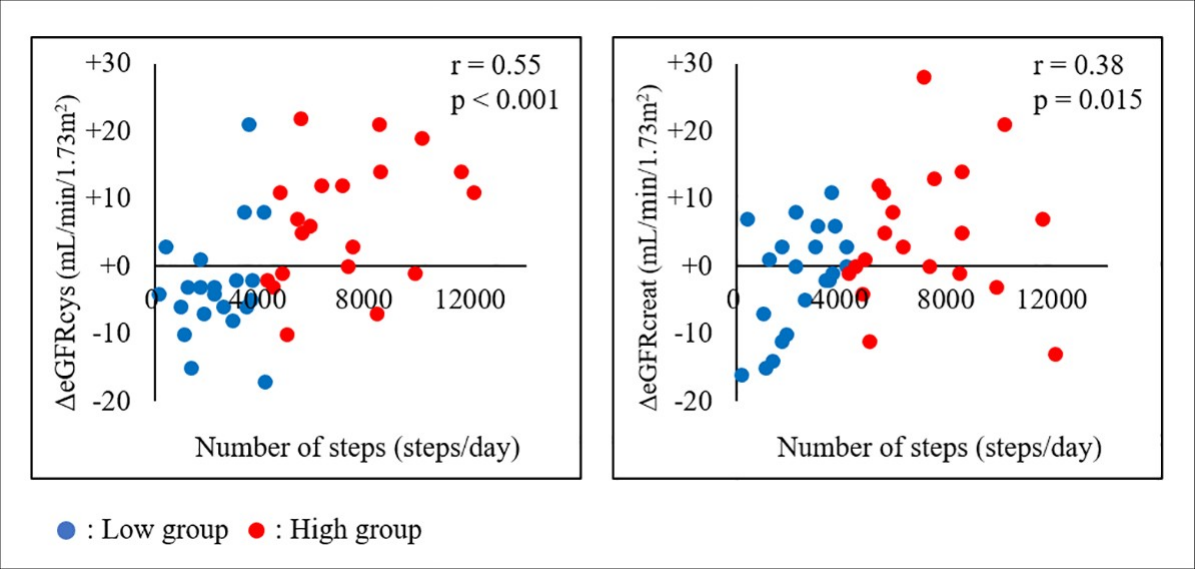
Associations between the number of steps and ΔeGFRcys or ΔeGFRcreat. eGFRcys, cystatin C-based estimated glomerular filtration rate; eGFRcreat, creatinine-based estimate glomerular filtration rate.

**Fig 2** shows the associations between the number of steps and ΔeGFRcys or ΔeGFRcreat in all patients. The results of Pearson’s correlation analysis showed significant correlations between the number of steps and both parameters. Furthermore, the correlation coefficient between ΔeGFRcreat and the number of steps (r = 0.38, p = 0.015) was lower compared to the correlation coefficient between ΔeGFRcys (r = 0.55, p < 0.001) and the number of steps.

## Discussion

The primary findings of this study are as follows: (1) physical activity level in daily life was positively associated with changes in renal function in patients after AMI, and (2) the number of steps correlated significantly and positively with both ΔeGFRcys and eGFRcreat in all patients, although the correlation coefficient using ΔeGFRcreat as an indicator for changes in renal function was lower than that using ΔeGFRcys.

In recent years, cardiac rehabilitation focusing mainly on supervised exercise therapy for patients with AMI [7] or a group of patients with cardiovascular disease [8,9,29,30] was found to maintain or improve renal function. According to Takaya et al., in a phase II cardiac rehabilitation program for 3 months for AMI patients, eGFRcreat improved significantly in active patients with a participation frequency of 1.1 times or more per week, but no significant improvement was observed in inactive patients with a participation frequency of less than 1.1 times per week [7]. There is a possibility that renal functions in AMI patients will improve with exercise therapy, and in such cases, maintaining the frequency and amount of exercise is especially important. However, most previous studies that examined the effect of exercise on renal function in AMI or CVD patients estimated renal function with serum creatinine, which depends on skeletal muscle mass [12]. Only one study [9] demonstrated that the renal function assessed by cystatin C improved in CVD patients with CKD after cardiac rehabilitation, but the study did not have a control group and was unable to verify the effect of amount of exercise on renal function.

In the present study, the physical activity of AMI patients was recorded from the time they woke up until bedtime for over 3 months using an accelerometer to verify the association with changes in the renal function over time. Additionally, we considered eGFRcys as a valid indicator of renal function in this study. The results indicated significant longitudinal associations between physical activity level and within-patient changes in eGFRcys, and in the analysis adjusted for potential confounding variables [23–27], similar significant association was confirmed. Additionally, exacerbation in the urine analysis data was not confirmed within each group as well. These results support and supplement prior studies [7–9] mentioned above that indicated the improving effect of exercise on renal function in AMI patients.

Significant correlations between changes in renal function and number of steps were found in all patients in the secondary analysis. However, the correlation coefficient using ΔeGFRcreat as an indicator for the changes in renal function was lower than that using ΔeGFRcys. Based on the scatter diagram (**Fig 2**), an increase in the variation of ΔeGFRcreat with the increase in the number of steps was confirmed. As previously pointed out, changes in the serum creatinine level through movement of the skeletal muscles [12,13] is one of the causes, and the significance of using eGFRcys as an indicator for renal function in this study was confirmed.

In recent years, a prior prospective study [31] verified the association between physical activity level and renal function in CKD patients. The results of this study are similar to our present findings and indicate that maintaining a high level of physical activity in daily life leads to suppression of renal function deterioration. In contrast, studies conducted on CKD patients have shown conflicting findings. A study [32] reported the same results as found in CKD patients, but a study also reported [33] that physical activity level does not affect renal function. Thus, the relationship between physical activity level and renal function is not clear for persons without CKD. Not being able to sufficiently grasp the level of physical activities using the previous methods may be one of the reasons. These reports were based on the evaluation of physical activity level, and measurements were conducted using a recollection type questionnaire to simplify the evaluation process. However, when elderly people are included in such studies, the evaluation results are possibly biased due to poor memory or inability to understand questions [34], and this may be one of the factors for conflicting results between studies.

In this study, reliability of the evaluation was ensured by measuring the amount of physical activity using an accelerometer. The adherence rate for the physical activity monitoring was 83.6% during the 3 months follow-up, and there were no missing data during the 14 days extracted for analysis. Additionally, the target patients of this study did not engage in exercises such as cycling or swimming, which cannot be measured using an accelerometer, during the observation period. The physical activity level measured in this study was a better reflection of the actual physical activity level in daily life.

This study is the first to show the association between physical activity level and changes in renal function after the onset of AMI, and it demonstrated that maintaining a high physical activity level is essential for protecting the renal function of AMI patients. The strengths of this study include the reliability of evaluating renal function and physical activity. In this study, the primary evaluation for estimating renal function utilized the cystatin C level, which has been reported as a more reliable marker of renal function than creatinine [12,13]. Furthermore, we analyzed the actual amount of physical activity by measuring the daily number of steps for 3 months using an accelerometer rather than a recollection type questionnaire, which may be associated with recall and other biases. In the clinical setting, the therapeutic strategy of using the number of steps in daily life, which is simple, cost-effective, and has less time constraints, may be easily accepted as one of the strategies to manage renal disease in outpatients after AMI. On the contrary, in this study, the clinical data at baseline indicated that the low group had significantly lower physical function than the high group. Furthermore, the differences similar to those observed at baseline between groups were observed even after 3 months. These results indicate that deterioration in the physical activity level was likely to occur continuously in patients with low physical function at baseline in a non-interventional situation. To increase physical activity and function of such patients, further intervention such as increasing the frequency of periodic follow-up after completion of phase I cardiac rehabilitation, providing education and counseling for physical activity [35], or continuing guidance on self-exercise with motivational coaching strategies and objective feedback on the training data [36] will be necessary.

This study has several limitations. First, it was a small, prospective, observational study without a control group, and most samples (85.4%) were men. For those reasons, all confounding factors that may affect the changes in the renal function were not sufficiently adjusted. We examined the control status of age, blood glucose level, lipid level, and blood pressure as background factors that may affect renal function. We analyzed these factors as covariates using GEE but did not confirm dietary intake status that includes management of salt and water intake because detailed evaluation after discharge of patients was difficult. Second, the verification of this study was limited to a follow-up period of 3 months. Whether the same results will be obtained in an extended observation period is unknown. Verifying whether the changes in renal function can be corrected by improving physical activity and physical function during the study is important, but this was not investigated in this study. For this reason, future research should verify the long-term effect of physical activity level on renal function among AMI patients in a larger cohort.

## Conclusion

The present study is the first to show the association between physical activity level and changes in renal function after the onset of AMI using an accelerometer and eGFRcys. High physical activity was suggested to suppress renal function decline in patients with AMI. Our findings support the importance of interventions to maintain high physical activity as a strategy for renal protection in AMI patients. Future research should verify the long-term effect of physical activity level on renal function among AMI patients.

## Supporting information

S1 File Data used for the analysis. (XLSX)
